# Correction: Atorvastatin inhibits Lipopolysaccharide (LPS)-induced vascular inflammation to protect endothelium by inducing Heme Oxygenase-1 (HO-1) expression

**DOI:** 10.1371/journal.pone.0320078

**Published:** 2025-03-13

**Authors:** Jian Luo, Qian Zhu, Keming Huang, Xue Wen, Yongquan Peng, Gang Wei, Gong Chen

The authors are listed out of order. Please view the correct author order, affiliations, and citation here:

Jian Luo^1^, Qian Zhu^1^, Keming Huang^1^, Xue Wen^2^, Yongquan Peng^1^, Gang Wei^1^, Gong Chen^1^

**1** Department of Cardiology, The Affiliated Hospital of Southwest Medical University, Lu Zhou, China, **2** Department of Laboratory Medicine, The Affiliated Hospital of Southwest Medical University, Lu Zhou, China

Luo J, Zhu Q, Huang K, Wen X, Peng Y, Wei G, et al. (2024) Atorvastatin inhibits Lipopolysaccharide (LPS)-induced vascular inflammation to protect endothelium by inducing Heme Oxygenase-1 (HO-1) expression. PLoS ONE 19(8): e0308823. https://doi.org/10.1371/journal.pone.0308823.
