# Supplementary material for: Atorvastatin inhibits Lipopolysaccharide (LPS)-induced vascular inflammation to protect endothelium by inducing Heme Oxygenase-1 (HO-1) expression
Source: PLoS One. 2024 Aug 15;19(8):e0308823. doi: 10.1371/journal.pone.0308823 (PMC11326635; doi:10.1371/journal.pone.0308823)
Supplement: S1 Table — (DOCX) [file pone.0308823.s001.docx]

**S1 table**

| **Serum bilirubin , ALT and TC concentrations in each group** | | | |
| --- | --- | --- | --- |
| Groups | Bilirubin（μmol/L） | ALT（U/L） | TC（mmol/L） |
| Control group | 7.04±0.69 | 96.60±6.02 | 1.71±0.13 |
| Low-dose atorvastatin group | 7.94±0.60* | 108.91±7.56* | 1.63±0.13 |
| High-dose atorvastatin group | 8.88±0.78** | 127.91±8.83** | 1.61±0.14 |
| HO-1 blocking group | 7.19±0.49 | 91.55±5.93 | 1.64±0.10 |

S1 table shows serum bilirubin , ALT and TC concentrations in four groups of mice in the experiment.* P < 0.05 compared with the control group; ** P < 0.05 compared with the control group.
