# Supplementary material for: Atorvastatin inhibits Lipopolysaccharide (LPS)-induced vascular inflammation to protect endothelium by inducing Heme Oxygenase-1 (HO-1) expression
Source: PLoS One. 2024 Aug 15;19(8):e0308823. doi: 10.1371/journal.pone.0308823 (PMC11326635; doi:10.1371/journal.pone.0308823)
Supplement: S2 Table — (DOCX) [file pone.0308823.s002.docx]

S2 Table

| **Concentrations of serum MDA, ET-1, NO, vWF, sTM, and EPCR in each group** | | | | | | |  |
| --- | --- | --- | --- | --- | --- | --- | --- |
| Groups | MDA | ET-1 | NO | vWF | sTM | EPCR |  |
|  | （nmol/ml） | （ng/l） | （μmol/l） | （ng/ml） | （ng/ml） | （ng/ml） |  |
| Control group | 9.03±0.71 | 113.69±4.21 | 25.23±2.01 | 3.93±0.28 | 5.38±0.18 | 4.70±0.22 |  |
|  |  |  |  |  |  |  |  |
| Low-dose atorvastatin group | 7.80±0.78* | 94.39±3.57** | 50.41±4.76** | 3.49±0.26** | 5.06±0.19** | 4.40±0.21* |  |
|  |  |  |  |  |  |  |  |
| High-dose atorvastatin group | 6.85±0.50** | 74.74±5.38** | 50.05±3.02* | 3.29±0.12** | 4.69±0.14** | 3.94±0.14** |  |
|  |  |  |  |  |  |  |  |
| HO-1 blocking group | 9.46±0.99 | 117.10±4.39 | 45.05±4.55 | 4.19±0.19 | 5.55±0.13 | 4.84±0.20 |  |
|  |  |  |  |  |  |  |  |

S2 Table shows serum MDA, ET-1, NO, vWF, sTM, and EPCR concentrations in 4 groups of mice in the experimen.* P < 0.05 compared with the control group; ** P < 0.05 compared with the control group.
