# Supplementary material for: Atorvastatin inhibits Lipopolysaccharide (LPS)-induced vascular inflammation to protect endothelium by inducing Heme Oxygenase-1 (HO-1) expression
Source: PLoS One. 2024 Aug 15;19(8):e0308823. doi: 10.1371/journal.pone.0308823 (PMC11326635; doi:10.1371/journal.pone.0308823)
Supplement: Attachment — Submitted filename: Reviewer Attachment.docx [file pone.0308823.s007.docx]

1. **Is the manuscript technically sound, and do the data support the conclusion?**

Yes

- **Strength**
- Technically it sounds good, the outcome is tested twice with CEC – circulating endothelial cells count and for tissue from direct descending aorta and both results are in coherence.
- For quantitative analysis of protein expression in the tissue the Coomassie Brilliant Blue method provides visual confirmation but may lack quantitative accuracy, but using high-power fields and high expertise could minimize the error. The ten 400x high power field where ten separate fields of view observed at a magnification of 400 times can ensure representative sampling of the tissue to increase the statistical robustness of the findings and it is enough as their tissue homogenate is homogenous.
- Typical sample size for experimental groups of Sprague-Dawley rats ranges from 8-12, of course it depends on statistical power, scientific validity and ethical consideration. The 10 sample size for each of four groups is enough sample size for this study. Controls are also used reverse control also being done by blocking HO-1 expression.
- The result, discussion and conclusion are inline and appropriate
- The biomarkers for endothelial injury and damage selected here (endothelial cell protein c receptors, endothelin-1, von Willbrand factor, and soluble thrombomodulin are appropriate, and others to measure the effect of atorvastatin such as HMG-CoA reductase for cholesterol TC, HO-1 expression and the LPS inflicted damage to the endothelial is also appropriate. The mechanism underlying the effect or atorvastatin through HMG-G inhibition and increasing HO-1 expression is also well explained.
- **To be clarified or corrected**
- The dosage of atorvastatin for low and high is 2 and 10 mg/kg/d. In researches involving Sprague-Dawley rats the dosage of atorvastatin; typically, or approximately range for low dose 1-5, medium 5-20 and high is above 20 mg/kg/day, here as the dose can vary based on specific research objective (here is to test high dose), duration of the study and weight of rats; it would be better if you mention the reason why you select the 2 and 10 mg dosage per kg/d, what is your background or could you reason out? Have you considered previous literature or do you have any dose-response studies to determine the most appropriate doses?

2. **Has the statistical analysis been performed appropriately and rigorously?**

Yes,

Strength

- one-way ANOVA and students t-test is used as their data was parametric, that is correct to compare the 3 or 2 groups, the right statistical methods used.

To be clarified or corrected

- Better to put the actual p value along with the cutting value 0.05 to get a highlight of how much it is significant in the result section instead of putting only like <0.05 or >0.05
- instead of saying the normally or approximately normally you should put the nature of distribution of your data aptly as normal or not or with the other words like parametric or non-parametric – this makes your analysis ambiguous so avoid the saying approximately here.
- In addition to this if multiple doses of atorvastatin are tested here, is it not possible to analyze dose-response relationships using regression analysis or non-linear modeling techniques– this is optional if you add this it will strengthen your study
- at what CI was the P value calculated it should have to be mentioned in the statistical methodology part at least once

3. **Have the authors made all data underlying the findings in their manuscript fully available?**

- No
- The average measurements were presented in the figures at the apex. The descriptive statistics part should be presented as additional data; the means, medians and variance measures should be available, according to PLOS data policy. It is better to be included here.

4. **Is the manuscript presented in an intelligible fashion and written in standard English?**

**Yes**

**Strength**

- The manuscript is presented with clear and standard English.

**To be corrected or clarified**

- The title “ ..is significantly in mitigating …“ is grammatically incorrect, the adjective significantly is used as a noun here. Use it as significant or if you prefer to use significantly you need to put it with its subordinate word such as high.– the third option is to restructure or change the word.
- In the introduction section “increased secretion of endothelin-1 (ET-1) from cells and a noticeable increase in cytokine levels, including malondialhdehyde (MDA), endothelial cell protein C receptor (EPCR), and ” in this sentence you should avoid including as it misleads that MDA is also a cytokine which it is not.
- In the abstract –“ based on heme oxygenase “ should be “ based on heme oxygenase expression”
- 1.3 statistical analysis
  - “The normally or approximately normally distributed data” – this phrase has grammatical error instead of saying the normally you need to say the normal or approximately normal, this comment is from the language perspective but statistically you need to be apt, if it is parametric just say it is parametric and if not just put non-parametric .
  - “meanSEM.One-way” – no space after full stop
  - The confidence level should also be mentioned along with the p value results at least once
- 1.2.4
  - “..CD31 positive cells.” Put the full stop.
- 2.2
  - “following trend(Figure 2)” need to be following trend (Figure 2)

5. **Review comments to the Author**

- Generally, it is interesting to review a technically rich and thoroughly made study like this. The Authors has put great effort to make a significant contribution to the scientific community and to the existing body of knowledge. Technically the study is rich and the presentation also so much interesting. I am happy to read their manuscript to review it. It is well done and keep it up guys.
